# Changes in Maxillary Sinus Mucosal Thickening following the Extraction of Teeth with Advanced Periodontal Disease: A Retrospective Study Using Cone-Beam Computed Tomography

**DOI:** 10.1155/2021/6688634

**Published:** 2021-03-23

**Authors:** Zheng Cao, Jie Yuan

**Affiliations:** Dental Clinic of Sir Run Run Shaw Hospital, School of Medicine, Zhejiang University, China

## Abstract

**Objectives:**

To explore the alterations in maxillary sinus mucosal thickening after extracting teeth with severe periodontal disease using cone-beam computed tomography (CBCT).

**Methods:**

30 patients with severe periodontal disease of maxillary posterior teeth that needed to be extracted and who were radiographically diagnosed with mucosal thickening (MT) in the maxillary sinus participated in the study. CBCT scans were taken before tooth extraction and 2-29 months after tooth extraction. The postextraction follow-up time was divided into two groups: group 1 (<4 months) and group 2 (≥4 months). Dimensions of maxillary sinus MT, including the MT zone length (SL) and the maximum thickness of the MT zone (ST), were evaluated preextraction and postextraction; the residual ridge height (RRH) was evaluated at the sites of extracted and nonextracted teeth.

**Result:**

Of the 24 patients with unilateral tooth extraction, there was a statistically significant difference in MT between the extraction and nonextraction sides (*p* < 0.05). The RRH at the site of the extracted teeth was significantly lower than that of the nonextracted teeth (*p* < 0.05). MT decreased significantly after tooth extraction on the extraction side but not on the nonextraction side. There was no significant difference between group 1 and group 2 regarding the reduction in mucosal thickness over time.

**Conclusions:**

Severe periodontitis can cause MT in the maxillary sinus. The RRH was lower at the sites of extracted teeth. MT reduced quickly by a thorough debridement after tooth extraction in 4 months. MT will not decrease further over time.

## 1. Introduction

With the popularity and development of dental implants, an increasing number of people are using cone-beam computed tomography (CBCT) for preoperative evaluation; consequently, mucosal thickening (MT) is found in symptomless maxillary sinuses. Though patients may be asymptomatic, there may still be clinical or pathological changes. The maxillary sinus membrane called the Schneiderian membrane is a thin respiratory mucous membrane that lines the maxillary sinus cavity. It adheres firmly to the periosteum and is approximately 0.8–1 mm thick [1, 2]. Radiographic membrane thickness was in agreement with clinical measurements of the human maxillary sinus membrane [3, 4]. When it is infected or irritated by an allergic reaction, the thickness increases 10-15 times. Therefore, a thickened sinus membrane is one of the important characteristics of maxillary sinus mucosa infection or stimulation. MT > 2 mm is reported to indicate maxillary sinusitis [5, 6]. There are many factors influencing MT, such as infection (caused by bacterial, fungal, or viral agents), foreign bodies, trauma, allergies, and immune deficiency [7, 8]. Other studies have shown that patient-related factors may also influence the thickness of the MT. Patients older than 60 years and smokers tend to have thicker mucous membranes [9].

Maxillary sinusitis is odontogenic and nasogenic, and the incidence of odontogenic maxillary sinusitis is 10%-12% [10]. Since the maxillary molars are adjacent to the maxillary sinus, the bacteria involved in the root tip area can easily enter the maxillary sinus through the porous alveolar bone, thus causing a maxillary sinus mucosal inflammatory response. There are several odontogenic pathogenic factors, such as periapical periodontitis, periodontitis, tooth fracture infection, bone grafts, dental implants, and iatrogenesis [10, 11]. Among them, periodontal disease is the most common disease as it has the highest incidence. In China, according to “The Fourth National Oral Health Epidemiological Report in 2017”, the periodontal health rate was only 9.1% [12]. It is a chronic infectious disease caused by plaque microorganisms, which often causes severe alveolar bone absorption in the late stage [8]. Due to serious infection, it is more likely to cause MT and even maxillary sinusitis.

Advanced periodontal disease could result in massive resorption of the alveolar. If there is not enough alveolar left in the posterior maxilla, it is not easy for implant placement; therefore, sinus floor elevation techniques should be considered [13–15]. However, some complications such as mucosal perforation, bleeding, and inflammation may occur during the surgical techniques. MT may be important factors for mucosal perforation that could lead to an increased rate of implant failure [16].

There are some studies on the relationship between periodontal disease and MT [17–20]. However, there are also some controversies. Some studies have shown that periodontal disease causes MT in the maxillary sinus, while others have suggested that there is no correlation. Ren et al. [17] studied 221 patients with periodontal disease and demonstrated positive correlations of MT and periodontitis. Phothikhun et al. [18] reported that sinuses with severe periodontal bone loss were three times more likely to have MT. However, in contrast to these results, Ramanauskaite et al. [19] evaluated a total of 414 CBCT images of 207 patients and presented no correlation between MT and the amount of periodontal bone loss. Shanbhag et al. [20] reported the same conclusion.

Most of the present studies are cross-sectional observation models. In recent years, there have been few relevant longitudinal experimental studies reported in the literature. In addition, almost all of the previous studies were performed when the periodontal teeth were not extracted. The present study is a longitudinal experimental study by extracting periodontally compromised teeth. The aims of the study were as follows: (1) to compare the difference in MT with and without the periodontally compromised teeth, (2) to compare the change in MT before and after the extraction of periodontally compromised teeth, (3) to compare the change in MT at two follow-up times (<4 months and ≥4 months), and (4) to compare the RRH at the site of the extracted and nonextracted teeth.

## 2. Method

The present study included 30 patients (21 males and 9 females; age range, 30 to 71 years; mean age: 53.17 ± 11.00 years). Patients who had at least 1 maxillary posterior tooth that needed to be extracted due to severe periodontal disease that was accompanied by thickening of the maxillary sinus mucosa were enrolled. Of the 30 patients, 24 were with unilateral tooth extraction and 6 with bilateral tooth extraction. All the patients had plans for implant-retained prostheses later, and they returned 2-29 months after tooth extraction.

All operations were accomplished at the Periodontology and Implantology of the Dental Department of Sir Run Run Shaw Hospital Affiliated with Zhejiang University, School of Medicine, from July 2016 to July 2020. Patients were asked to sign an informed consent form before tooth extraction. This retrospective radiologic study was approved by the Ethics Committee of Sir Run Run Shaw Hospital Affiliated with Zhejiang University School of Medicine (No. 20190916-11) in full accordance with the World Medical Association Declaration of Helsinki.

### 2.1. Inclusion Criteria

1. Over 18 years of age
2. Presence of at least 1 posterior maxillary tooth requiring extraction due to advanced periodontal disease, as assessed by a probing pocket depth (PPD) of ≥6 mm, clinical attachment level (CAL) of ≥5 mm, and an imaging examination showing severe periodontal bone loss as indicated by more than 50% bone loss of the total root length, which could detect root bifurcation lesions and tooth looseness ≥ II
3. Presence of local mucous MT in the maxillary sinus > 2 mm corresponding to the extracted tooth site; thickened mucosal margin and the floor or the wall of the sinus intersected at an angle of <30° [21]
4. Good sinus visibility on the CBCT scan

### 2.2. Exclusion Criteria

1. Pregnant or nursing women
2. Common cold or sinusitis in the past months
3. Asthma or allergic rhinitis
4. Mucosal lesions that could be diagnosed as mucous retention cysts, polyposis, or tumors
5. Periapical lesions in the regions studied
6. Acute inflammation in the tooth

### 2.3. Clinical Procedures

All the patients had at least one tooth to be extracted and had plans for implant-retained prostheses later. A total of 58 hopeless teeth with advanced periodontal disease were extracted. Patients underwent a professional oral examination to evaluate the state of dentition, clinical periodontal parameters such as probing depth, bleeding on probing, tooth mobility, and risk factors. A comprehensive plan for periodontal treatment was drawn. Following oral hygiene instruction, necessary initial periodontal therapy was provided. CBCT scans were conducted and were used to establish correct periodontal diagnoses as well to provide appropriate treatment options.

Patients were asked to rinse with 0.2% chlorhexidine mouthwash for 30 seconds. Tooth extraction was completed in a minimally traumatic fashion under local anesthesia (4% articaine with adrenaline 1 : 100,000). Then, the socket was scratched thoroughly to remove inflammatory granulation tissue and infective lesions. The patient did not take any antibiotics after tooth extraction. The second set of CBCT scans was conducted 2–29 months after tooth extraction according to the time of patient implant schedule.

### 2.4. Imaging Procedure

All images were acquired using a 3D CBCT scanner (KODAK CS 3D Imaging System, Carestream Health, Inc., France). Operating parameters were set at 10.0 mA and 90 kV, and the exposure time was 10.8 seconds. Slices at 0.30 mm intervals were reconstructed on the sagittal and coronal planes for evaluation and measurements using inbuilt software (Kodak Imaging Software CS 3D Imaging; Carestream Health Inc.).

### 2.5. Assessment of Mucosal Thickening

**Figure 1:**
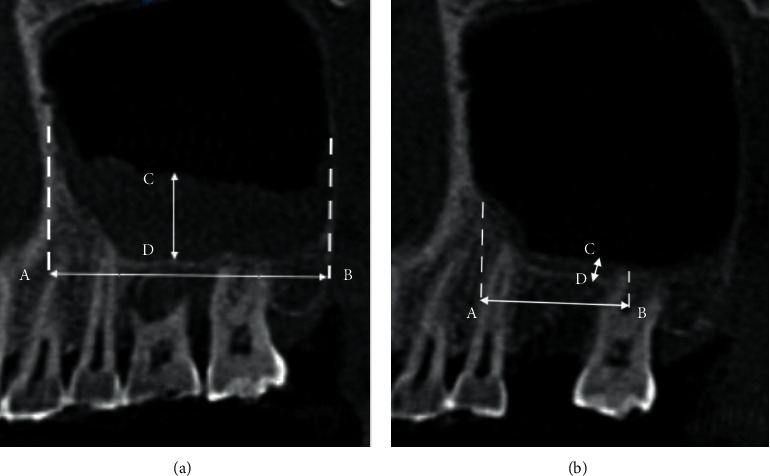
Sagittal section showing changes in SL and ST before and after tooth extraction in patients with MT. (a) Before tooth extraction. (b) Four months after tooth extraction. SL is the distance from the beginning to the end of the total area of sinus mucosal thickening (that is, the distance between points A and B). ST is the distance between the sinus floor and the highest point of the mucosa in the total area exhibiting sinus mucosal thickening (that is, the distance between points C and D).

The measurement of all patients was taken as reference for the maxillary first molar. The axial plane was adjusted to the neck of the maxillary first molar, the coronal plane was adjusted to bisect the buccal and lingual sides of the maxillary first molar, and the sagittal plane was adjusted to bisect the proximal and distal surfaces of the maxillary first molar. If the maxillary first molar is missing, the adjacent tooth was selected. Images were analyzed and the data were measured in terms of SL and ST. The length of the sinus MT (SL) was defined as the distance from the beginning to the end of the total area exhibiting sinus MT [22, 23], that is, the distance between points A and B. The thickness of the sinus MT (ST) was defined as the distance between the sinus floor and the highest point of the mucosa in the total area exhibiting sinus MT [22, 23], that is, the distance between points C and D (Figure 1). MT was considered present when the mucosa thickness was >2 mm [24].

### 2.6. Assessment of Residual Ridge Height (RRH)

**Figure 2:**
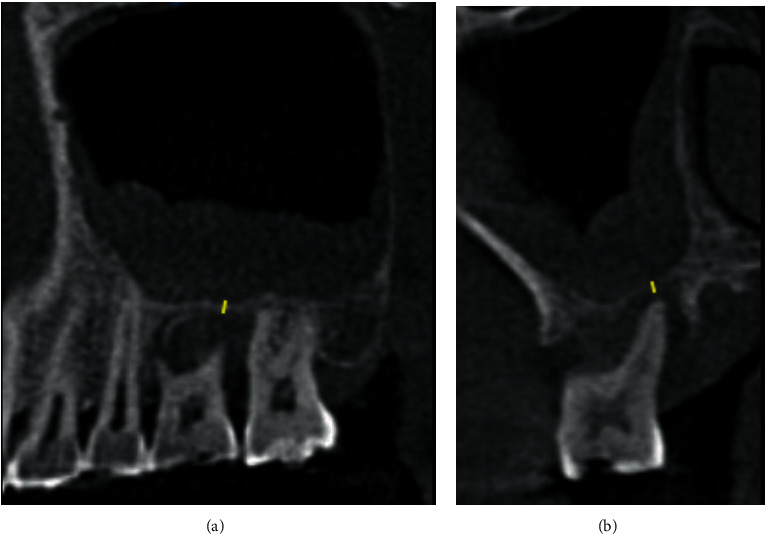
(a) Sagittal section showing the RRH of the extracted teeth. The yellow line shows the RRH. (b) Coronal section showing the RRH of the extracted teeth. The yellow line shows the RRH.

RRH was determined by scrolling through the scan in the coronal and sagittal planes of the extracted teeth and the nonextracted teeth. A reference line was drawn on the CBCT image from the most apical alveolar bone region of the periodontal lesion to the floor of the maxillary sinus [25, 26]. The RRH was shown in the yellow line in Figure 2.

### 2.7. Assessment of MT after Extraction

A second CBCT scan was taken 2–29 months after tooth extraction before implant placement. SL and ST data were measured again. According to Huang et al. [27], the recovery of maxillary sinus mucosa after sinus surgery in chronic sinusitis is 4 months. Thus, 4 months served as the standard cut-off. Two groups were categorized as follows: (1) <4 m and (2) ≥4 m.

### 2.8. Statistical Analysis

A research investigator made all the radiographic measurements. For the calibration and assessment of intraexaminer reliability, 10 randomly selected scans were measured twice with an interval of 7 days (mean difference = 0.02-0.05 mm). Intraexaminer agreement was determined as a Cohen kappa of 0.81 and 0.82, respectively.

All statistical analyses were performed by R Version 3.6.3 software. The descriptive analysis of the data was presented as frequencies for categorical variables and medians (interquartile ranges) for continuous variables. Associations between variables were evaluated using the Wilcoxon test. *p* < 0.05 was considered statistically significant.

## 3. Results

During the study period, a total of 30 patients with 60 sinuses were included for analysis of changes in MT based on their CBCT images. Each patient had at least one sinus with at least one tooth extraction. Of the 30 patients, 24 were with unilateral tooth extraction and 6 with bilateral tooth extraction. Of the 60 sinuses, 36 were with tooth extraction and 24 with nonextraction. We analyzed 152 teeth and 58 of them were extracted. The distribution of tooth extraction shows that most of them were the first and second molars (22 first molars and 21 second molars). MT was present in 49 sinuses (81.7%), with 35 exhibiting MT on the tooth extraction side (71.4%) and 14 exhibiting MT on the nonextraction side (28.6%). After tooth extraction, the patients who had an implant plan returned after 2 to 29 months, with 10 returning in <4 months and 20 returning in ≥4 months. The clinical characteristics of the patients are shown in Table 1.

### 3.1. Assessment of Residual Ridge Heights (RRHs)

Of all 152 teeth under the maxillary sinus, 58 were extracted, and 94 were not extracted. The mean RRH in our study sample in the area of the extracted teeth was 1.40 (1.00-3.18) mm and 6.30 (4.80-9.48) mm in the area of the nonextracted teeth. RRH of the extracted tooth area was significantly lower than that of the nonextracted tooth area (*p* < 0.001).

### 3.2. Assessment of MT on the Tooth Extraction and Nonextraction Sides

Of all 30 patients, 24 presented unilateral tooth extraction. The median MT values were SL 25.90 (22.10-30.23) mm and ST 8.25 (5.30-10.03) mm on the extraction side and SL 18.15 (0-22.70) mm and ST 2.55 (0-5.98) mm on the nonextraction side. There was a statistically significant difference between the MT values (Table 2). Notably, greater MT was present on the tooth extraction side.

### 3.3. Assessment of MT before and after Tooth Extraction

Before tooth extraction, SL and ST significantly increased on the extraction side. After tooth extraction, the mean SL decreased from 25.90 (20.98-31.55) mm to 13.05 (0-18.38) mm, and the ST decreased from 7.55 (3.18-9.95) mm to 1.70 (0-2.43) mm. However, on the nonextraction side, there was no significant change in SL and ST before and after tooth extraction (Table 3).

The mucosal thickness distribution analysis showed that MT was found in 35 sinuses before extraction and 14 sinuses after extraction on the tooth extraction side. On the nonextraction side, MT was found in 14 sinuses before extraction and 14 sinuses after extraction (Table 3).

### 3.4. Assessment of Timing on the Change of MT after Tooth Extraction

Table 4 presents the values of the reduction in MT in different groups (group 1, <4 m; group 2, ≥4 m) on the tooth extraction and nonextraction sides. SLD = SL (follow‐up) − SL (baseline), STD = ST (follow‐up) − ST (baseline). There were no significant differences in SLD and STD between group 1 and group 2. There was no indication that the thickened sinus mucosa was to return to normal on the extraction side after more time passed.

## 4. Discussion

Our study demonstrated that severe periodontitis could cause MT in the maxillary sinus. We selected patients who had at least 1 maxillary posterior tooth that needed to be extracted due to severe periodontal disease and in which MT corresponding to the extracted tooth. Some factors that could cause MT, such as allergies, periapical lesions, and mucous retention cysts, were excluded. Moreover, all the patients were not treated with antibiotics, thus excluding the effect of drugs on MT.

In the present study, the tooth extraction position distribution shows that most of them were mainly first and second molars. Of all 58 teeth extracted, 43 were first and second molars, accounting for 74.1%. Hence, it seems that maxillary molars are more prone to alveolar bone resorption. Our results, in general, are consistent with those reported in previous studies. McFall et al. [28] reported that among periodontally compromised teeth, maxillary molars are the teeth most likely to be lost. The higher risk for maxillary molars may be explained by the fact that their complex morphology with multiple roots, root fusion or root proximity, and furcation entrances is difficult to access with self-performed oral hygiene [29]. However, MT not only occurred corresponding to the site of the extracted tooth but also occurred to some extent in the adjacent teeth without obvious alveolar resorption. It is supposed that pathogenic bacteria and their products around the extracted tooth infiltrate the porous sinus floor and blood vessels and lymphatic vessels to the sinus mucosa, resulting in spread of infection and extensive MT [30, 31].

Based on the measurements of the CBCT image, obvious MT was present on the side of the sinus that underwent tooth extraction compared with the side of nontooth extraction. The reason may be as follows. (1) An increase in the quantity of bacteria and toxins from teeth with periodontal disease results in an increase in the degree of MT. It has been shown that the level of pathogenic bacteria and bacterial products as well as inflammatory cytokines increased significantly at sites with severe periodontitis [32]. (2) Also, in patients with severe periodontitis, RRH is significantly reduced due to the severe destruction of the alveolar bone [33, 14], and inflammation is more likely to spread to the maxillary sinus. In our study, the RRH of the tooth extraction group was 1.40 (1.00-3.18) mm, while the RRH of the nonextraction group was 6.30 (4.80-9.48) mm. There was a significant difference between the two groups (*p* < 0.001). Yilmaz and Tözüm [16] reported a higher prevalence of MT in patients with RRH < 3.5 mm, and this study was consistent with theirs [16, 25].

At present, there is a lack of relevant research on the changes in MT with thorough debridement after tooth extraction. We found that the dimensions of MT decreased significantly with the elimination of granulation tissue. According to Shanbhag et al. [24], mucosal thickness is normal when it is ≤2 mm. It was found that before extraction, there were 35 maxillary sinuses with MT > 2 mm. It goes down to 14 sinuses with MT > 2 mm after extraction. This means that with the healing of inflamed periodontal tissues, a large number of pathogens and their products had nowhere to reside; thus, most areas with MT returned to normal.

In this study, the changes in MT in different time periods, <4 months and ≥4 months, after tooth extraction were analyzed. All patients had significantly decreased MT on the side of tooth extraction. There was a significant difference before and after tooth extraction, but no difference was found between the two groups. This meant there was no further decrease in MT as more time passed. This conclusion is different from Yoo et al.'s conclusion [34]. In their study, MT gradually decreased as more time passed and returned to normal in the >12-month group. This difference in outcome may be because Yoo et al.'s results were based on a retrospective analysis of existing CBCT. The cause of tooth extraction was obtained by history taking, and nothing was known about the condition of MT before tooth extraction, so accurate analysis could not be made. Our conclusion is the same as Hsu et al.'s conclusion [35] that MT could be normalized in an average time of 2.8 months after tooth extraction, but Hsu et al.'s study only evaluated 6 periodontal patients, and only radiographic mucosal changes were assessed. No clinical data regarding patients' sinusitis-related history or symptoms were analyzed, and the factors of nonodontogenic maxillary sinusitis were also unknown.

On the other hand, we found no significant difference on the nonextraction side between preextraction and postextraction. Additionally, no difference was found <4 months and ≥4 months after tooth extraction. With regard to the data relativeness of patients with bilateral tooth extraction, sensitivity analyses were performed after excluding 6 patients with bilateral tooth extraction in Tables 3 and 4; the findings did not alter. It can be inferred that tooth extraction could treat infections and result in radiographic reduction in the MT. However, the infection on the nonextraction side was not eliminated, resulting in no significant change in mucosal thickness. Therefore, a probable hypothesis is that untreated severe periodontal disease at the maxillary posterior tooth can initiate local pathological changes in the mucosa of the maxillary sinus.

In our study, it was found that MT could be normalized in 4 months after tooth extraction. With regard to the timing of implant placement, a conventional treatment protocol involving tooth extraction is for a period of >4 months, followed by implant placement in a healed ridge [36]. That means when it is time to implant, if the sinus-lift procedure should be considered, the MT has been normalized. MT caused by severe periodontal disease would not increase the probability of implant failure.

We often encounter patients with maxillary sinusitis who have recurrent episodes after several surgical treatment and long-term antibiotics to control the infection. Upon referral to the dentist, it was found that maxillary sinusitis was caused by odontogenic factors. Sinusitis can be completely resolved after the solution of odontogenic source. So determining the reason of sinusitis is very important for clinicians. At present, CBCT has become an appropriate imaging technique for sinus examination due to its high resolution, low radiation, and low cost [37]. Patients with maxillary sinus disease should be evaluated by CBCT imaging before treatment. If the odontogenic factors are found, the treatment will be different. In this study, MT in most patients was quickly solved by extracting the teeth without the administration of antibiotic treatment.

Due to the limited sample size in this study and because only CBCT imaging was used to observe the morphological changes of the maxillary sinus mucosa and due to the lack of microbiology and histopathological studies, further research should be conducted in combination with the above aspects in the future.

## 5. Conclusions

The findings of this study show that severe periodontitis can cause MT in the maxillary sinus. Additionally, the RRH was lower at the sites of extracted teeth. MT reduced quickly by a thorough debridement after tooth extraction in 4 months, and MT will not decrease further over time.

## Figures and Tables

**Table 1:** Characteristics of patients.

Characteristics	Patients	
Age (years)	53.17 ± 11.00	
Sex		
Female	9	30%
Male	21	70%
Number of patients	30	
With unilateral tooth extraction	24	80%
With bilateral tooth extraction	6	20%
Number of sinuses	60	
With tooth extraction	36	60%
With nonextraction	24	40%
Number of teeth	152	
Nonextraction tooth	94	61.8%
Extraction tooth	58	38.2%
Distribution of mucosal thickening		
Tooth extraction side	35	71.4%
Nonextraction side	14	28.6%

**Table 2:** Measurements of MT by CBCT at the tooth extraction side and the nonextraction side among patients with unilateral tooth extraction (*N* = 24).

Variables	Tooth extraction side	Nonextraction side	*p* value
SL (mm)	25.90 (22.10-30.23)	18.15 (0-22.70)	<0.001
ST (mm)	8.25 (5.30-10.03)	2.55 (0-5.98)	<0.001

SL: the length of the sinus mucosal thickening; ST: the thickness of the sinus mucosal thickening. Statistically significant differences (*p* < 0.05) were determined using the Wilcoxon test.

**Table 3:** Measurements of MT by CBCT on the tooth extraction side and the nonextraction side before and after tooth extraction.

Variables	Baseline	Follow-up	*p* value
Tooth extraction side (*N* = 36)			
SL (mm)	25.90 (20.98 -31.55)	13.05 (0-18.38)	<0.001
ST (mm)	7.55 (3.18-9.95)	1.70 (0-2.43)	<0.001
MT, *n* (%)	35 (97.2%)	14 (38.9%)	<0.001
Nonextraction side (*N* = 24)			
SL (mm)	18.15 (0-22.70)	19.00 (0-23.75)	0.142
ST (mm)	2.55 (0-5.98)	2.50 (0–7.03)	0.232
MT, *n* (%)	14 (58.3%)	14 (58.3%)	1

SL: the length of the sinus mucosal thickening; ST: the thickness of the sinus mucosal thickening. Statistically significant differences (*p* < 0.05) were determined using the Wilcoxon test.

**Table 4:** Measurements of MT changes by CBCT on the tooth extraction side and nonextraction side in the two groups.

Variables	Group 1, <4 months	Group 2, ≥4 months	*p* value
Tooth extraction side	*N* = 12	*N* = 24	
SLD (mm)	-14.85 (-21.38, -8.38)	-12.55 (-16.48, -4.65)	0.306
STD (mm)	-4.80 (-7.90, -2.85)	-3.40 (-7.38, -1.48)	0.535
Nonextraction side	*N* = 8	*N* = 16	
SLD (mm)	0.25 (-0.13, 0.75)	0 (-0.05, 0.63)	0.951
STD (mm)	0.05 (-0.10, 0.23)	0 (-0.03, 0.13)	0.950

SLD: SL (follow‐up) − SL (baseline); STD : ST (follow‐up) − ST (baseline). Statistically significant differences (*p* < 0.05) were determined using the Wilcoxon test.

## Data Availability

The data used to support the findings of this study are included within the supplementary information file(s).

## Supplementary Materials

Supplementary Materials Table 1: characteristics of the patients and measurements of MT by CBCT. Table 2: measurements of residual ridge height (mm) at the maxillary posterior region.
