# Supplementary material for: Changes in Maxillary Sinus Mucosal Thickening following the Extraction of Teeth with Advanced Periodontal Disease: A Retrospective Study Using Cone-Beam Computed Tomography
Source: Biomed Res Int. 2021 Mar 23;2021:6688634. doi: 10.1155/2021/6688634 (PMC8009702; doi:10.1155/2021/6688634)
Supplement: Supplementary Materials — Table 1: characteristics of the patients and measurements of MT by CBCT. Table 2: measurements of residual ridge height (mm) at the maxillary posterior region. [file 6688634.f1.docx]

**Table 1. Characteristics of the patients and** **Measurements of MT by CBCT**

| **Patients** | **Sex** | **Age** | **Before/After tooth extraction** | **Length of MT (mm)** | | **Thickness of MT (mm)** | | **Distribution of tooth extraction** | **Follow -up time (month)** |
| --- | --- | --- | --- | --- | --- | --- | --- | --- | --- |
|  |  |  |  | **Left sinus** | **Right sinus** | **Left sinus** | **Right sinus** |  |  |
| 1 | F | 32 | Before | 33 | 33.2 | 11 | 5.4 | 18, 27, 28 | <4 |
|  |  |  | After | 28 | 29 | 2.1 | 2.3 |  |  |
| 2 | M | 30 | Before | 0 | 25.9 | 0 | 8.1 | 16,17 | >4 |
|  |  |  | After | 0 | 0 | 0 | 0 |  |  |
| 3 | M | 54 | Before | 15 | 29.8 | 6.2 | 7.1 | 15, 18 | 4-12 |
|  |  |  | After | 14.3 | 14.5 | 6 | 4.8 |  |  |
| 4 | M | 58 | Before | 12 | 24 | 2.1 | 16 | 16, 17, 26, 27 | 4-12 |
|  |  |  | After | 0 | 21.6 | 0 | 10.2 |  |  |
| 5 | F | 49 | Before | 21 | 20 | 6 | 1.2 | 26 | <4 |
|  |  |  | After | 0 | 21 | 0 | 1.3 |  |  |
| 6 | M | 50 | Before | 29.5 | 17.4 | 9.6 | 2.9 | 26, 27 | <4 |
|  |  |  | After | 19 | 17.3 | 1.7 | 2.8 |  |  |
| 7 | F | 58 | Before | 0 | 17.4 | 0 | 2.1 | 16, 17 | <4 |
|  |  |  | After | 0 | 0 | 0 | 0 |  |  |
| 8 | F | 63 | Before | 25.9 | 0 | 5.7 | 0 | 24, 25, 27 | 4-12 |
|  |  |  | After | 0 | 0 | 0 | 0 |  |  |
| 9 | M | 33 | Before | 33 | 35.1 | 8.7 | 5.9 | 27 | <4 |
|  |  |  | After | 10.5 | 35.6 | 1.3 | 6.9 |  |  |
| 10 | F | 44 | Before | 15.9 | 25.9 | 10.4 | 8.4 | 15, 16, 17, 18 | >12 |
|  |  |  | After | 18.8 | 12.8 | 12.4 | 1.2 |  |  |
| 11 | M | 32 | Before | 18.9 | 31.8 | 9.3 | 11.3 | 17 | 12 |
|  |  |  | After | 19.2 | 0 | 9.5 | 0.5 |  |  |
| 12 | M | 48 | Before | 10.9 | 28.9 | 2.8 | 11.5 | 16, 26, 27 | 4-12 |
|  |  |  | After | 7 | 14.2 | 2.1 | 9.3 |  |  |
| 13 | M | 58 | Before | 26.5 | 30.3 | 16.6 | 18.1 | 26, 28 | 4-12 |
|  |  |  | After | 24.4 | 31 | 15.2 | 17.7 |  |  |
| 14 | M | 49 | Before | 20.8 | 0 | 2.9 | 0 | 25, 26 | 4-12 |
|  |  |  | After | 0 | 0 | 0 | 0 |  |  |
| 15 | M | 71 | Before | 27.0 | 20.9 | 4.3 | 5.9 | 15, 16 | >4 |
|  |  |  | After | 26.8 | 18.6 | 4.1 | 4.6 |  |  |
| 16 | M | 51 | Before | 29.2 | 20.3 | 14.1 | 6.3 | 26, 27 | >12 |
|  |  |  | After | 23.9 | 23.6 | 2.1 | 7.4 |  |  |
| 17 | F | 66 | Before | 23.9 | 13.9 | 8.9 | 1.2 | 25, 26, 27 | <4 |
|  |  |  | After | 8.3 | 13.7 | 1 | 1.3 |  |  |
| 18 | M | 54 | Before | 33.3 | 35.5 | 8.9 | 14.5 | 17 | >4 |
|  |  |  | After | 32.6 | 34.5 | 8.9 | 10.8 |  |  |
| 19 | M | 68 | Before | 22.4 | 21.6 | 4.1 | 5.9 | 28 | >12 |
|  |  |  | After | 14.4 | 24.2 | 3 | 9.8 |  |  |
| 20 | M | 57 | Before | 0 | 31.7 | 0 | 11.3 | 16, 17 | >12 |
|  |  |  | After | 0 | 0 | 0 | 0 |  |  |
| 21 | M | 56 | Before | 0 | 31.5 | 0 | 9.6 | 17 | 4-12 |
|  |  |  | After | 0 | 11.5 | 0 | 1.7 |  |  |
| 22 | F | 46 | Before | 13.6 | 15.3 | 1.5 | 2.2 | 17, 27 | 4-12 |
|  |  |  | After | 0 | 11.4 | 0 | 0.8 |  |  |
| 23 | M | 61 | Before | 24.2 | 23.6 | 2.9 | 4.5 | 26 | <4 |
|  |  |  | After | 14.7 | 24.5 | 1.7 | 5.1 |  |  |
| 24 | F | 56 | Before | 24.2 | 21.2 | 2.1 | 3.2 | 16 | 4-12 |
|  |  |  | After | 22.9 | 13.5 | 2.2 | 2.8 |  |  |
| 25 | M | 69 | Before | 19.4 | 30.9 | 6 | 8.3 | 16, 17, 26 | >12 |
|  |  |  | After | 13.3 | 17.4 | 2.2 | 1.9 |  |  |
| 26 | M | 64 | Before | 22.4 | 23.9 | 2.2 | 2.1 | 16 | <4 |
|  |  |  | After | 23.1 | 22.7 | 2.1 | 1.8 |  |  |
| 27 | M | 65 | Before | 21.5 | 32.4 | 3 | 21.7 | 17, 18 | <4 |
|  |  |  | After | 20.8 | 18.3 | 2.9 | 1.8 |  |  |
| 28 | M | 51 | Before | 29.8 | 4.7 | 9.6 | 1.9 | 26 | 4-12 |
|  |  |  | After | 14.7 | 5.3 | 1.5 | 1.8 |  |  |
| 29 | M | 48 | Before | 32.1 | 35.7 | 3.6 | 3.1 | 17, 24 | <4 |
|  |  |  | After | 0 | 0 | 0 | 0 |  |  |
| 30 | F | 53 | Before | 0 | 17.2 | 0 | 8 | 16 | 4-12 |
|  |  |  | After | 0 | 12.3 | 0 | 4.9 |  |  |

**Table 2.** **Measurements of Residual ridge height (mm) at the maxillary posterior region**

| **Patients** | **Sex** | **Age** | **Residual ridge height (mm) at the maxillary posterior region** | | | | | | | | | | **Distribution of tooth extraction** |
| --- | --- | --- | --- | --- | --- | --- | --- | --- | --- | --- | --- | --- | --- |
|  |  |  | **#18** | **#17** | **#16** | **#15** | **#14** | **#28** | **#27** | **#26** | **#25** | **#24** |  |
| 1 | F | 32 | 1.5 | 6.1 | 4.3 | 7.1 |  | 2.7 | 0 | 1 | 4.8 |  | 18，27，28 |
| 2 | M | 31 | 4.1 | 1 | 1 | 23 |  | 8.3 | 7.1 | 6.3 | 18.5 |  | 16，17 |
| 3 | M | 54 | 3.6 | 5.6 |  | 3.1 |  |  |  |  | 7.6 |  | 15，18 |
| 4 | M | 58 |  | 1 | 1 |  |  |  | 3.8 | 6.1 |  |  | 16，17，26，27 |
| 5 | F | 49 |  | 7.9 | 6.1 | 12 |  |  | 4.8 | 1 | 9.6 |  | 26 |
| 6 | M | 50 |  |  | 2.4 | 7.8 |  |  | 1 | 0 |  |  | 26，27 |
| 7 | F | 58 |  | 0.8 | 2 |  |  |  | 7.8 | 6.8 |  |  | 16，17 |
| 8 | F | 63 | 8 | 3.6 | 3.2 |  | 6.2 | 2.4 | 1.2 |  | 0 | 1.3 | 24，25，27 |
| 9 | M | 33 |  | 5.2 | 6.2 | 9.8 |  |  | 2.1 |  | 5.3 |  | 27 |
| 10 | F | 44 | 1 | 1 | 2 | 1.5 |  |  |  | 2.2 | 11 |  | 15，16，17，18 |
| 11 | M | 32 |  | 1.5 |  | 8.2 |  |  | 4.8 | 3.6 | 7 |  | 17 |
| 12 | M | 48 |  | 5.8 | 4.1 | 15 |  |  | 6.4 | 7.8 | 17 |  | 16，26，27 |
| 13 | M | 58 |  | 5.1 | 6.7 |  |  | 1.2 |  | 1.5 |  |  | 26，28 |
| 14 | M | 49 |  | 6.1 |  |  |  |  | 5.1 | 3.2 | 3.7 |  | 25，26 |
| 15 | M | 71 |  |  | 1.1 | 3.7 |  | 6.2 |  |  |  |  | 15，16 |
| 16 | M | 51 |  |  |  |  |  |  | 2.1 | 1.8 | 9.5 |  | 26，27 |
| 17 | F | 66 |  | 5.3 | 4.7 | 7.9 |  |  | 0 | 0.5 | 6.5 |  | 25，26，27 |
| 18 | M | 54 |  | 1.1 | 1.2 | 6.1 |  | 2 | 1.2 | 2.4 | 4.6 |  | 17 |
| 19 | M | 68 |  | 5.6 | 11 |  |  | 3 | 9.3 | 3.6 |  |  | 28 |
| 20 | M | 57 |  | 0.7 | 1.6 | 6.3 |  |  |  |  | 5.1 |  | 16，17 |
| 21 | M | 56 |  | 1.1 | 1.2 | 14 |  |  | 5.8 |  | 9.2 |  | 17 |
| 22 | F | 46 | 9.4 | 2.4 | 1.5 | 9.8 |  | 3.7 | 1.1 | 13 |  |  | 17，27 |
| 23 | M | 61 |  | 4.3 | 6.4 | 18 |  |  | 12.4 | 5.5 | 14 |  | 26 |
| 24 | F | 56 |  |  | 5.3 | 14 |  |  | 12 | 12 | 14 |  | 16 |
| 25 | M | 69 |  | 1.2 | 0.8 | 10 |  |  |  | 0.8 | 8.8 |  | 16，17，26 |
| 26 | M | 64 |  | 16 | 10 | 9.8 |  |  |  |  |  |  | 16 |
| 27 | M | 65 | 3.5 | 1 |  |  |  |  |  |  |  |  | 17，18 |
| 28 | M | 51 |  |  | 3.9 | 7.7 |  |  | 3.4 | 1 | 11 |  | 26 |
| 29 | M | 48 |  | 1.2 |  | 5.1 |  |  | 4.4 |  | 5.3 | 5.2 | 17，24 |
| 30 | F | 53 |  |  | 1.1 | 8.9 |  |  | 4.9 | 6.3 | 6.4 |  | 16 |

*
